# Dataset of 143 metagenome-assembled genomes from the Arctic and Atlantic Oceans, including 21 for eukaryotic organisms

**DOI:** 10.1016/j.dib.2023.108990

**Published:** 2023-02-15

**Authors:** Anthony Duncan, Kerrie Barry, Chris Daum, Emiley Eloe-Fadrosh, Simon Roux, Katrin Schmidt, Susannah G. Tringe, Klaus U. Valentin, Neha Varghese, Asaf Salamov, Igor V. Grigoriev, Richard M. Leggett, Vincent Moulton, Thomas Mock

**Affiliations:** a School of Computing Sciences, University of East Anglia, Norwich Research Park, Norwich, NR47TJ, UK; b US Department of Energy Joint Genome Institute, 1 Cyclotron Road, Berkeley, CA, 94720, USA; c School of Environmental Sciences, University of East Anglia, Norwich Research Park, Norwich, NR47TJ, UK; d Alfred-Wegener Institute for Polar and Marine Research, Am Handelshafen 12, 27570, Bremerhaven, Germany; e Earlham Institute, Norwich Research Park, Norwich, NR4 7UG, UK

**Keywords:** Genome resolved metagenomics, Phycology, Marine algae, MAGs, Phytoplankton, Polar microbiomes

## Abstract

This article presents metagenome-assembled genomes (MAGs) for both eukaryotic and prokaryotic organisms originating from the Arctic and Atlantic oceans, along with gene prediction and functional annotation for MAGs from both domains. Eleven samples from the chlorophyll-a maximum layer of the surface ocean were collected during two cruises in 2012; six from the Arctic in June-July on ARK-XXVII/1 (PS80), and five from the Atlantic in November on ANT-XXIX/1 (PS81). Sequencing and assembly was carried out by the Joint Genome Institute (JGI), who provide annotation of the assembled sequences, and 122 MAGs for prokaryotic organisms. A subsequent binning process identified 21 MAGs for eukaryotic organisms, mostly identified as Mamiellophyceae or Bacillariophyceae. The data for each MAG includes sequences in FASTA format, and tables of functional annotation of genes. For eukaryotic MAGs, transcript and protein sequences for predicted genes are available. A spreadsheet is provided summarising quality measures and taxonomic classifications for each MAG. These data provide draft genomes for uncultured marine microbes, including some of the first MAGs for polar eukaryotes, and can provide reference genetic data for these environments, or used in genomics-based comparison between environments.

**Specifications Table**

**Table:** 

Subject	Microbiology: Microbiome		
Specific subject area	Surface ocean microbial communities		
Type of data	FASTA files Tables		
How the data were acquired	Seawater samples were sequenced using Illumina HiSeq platform, generating paired end 2 × 150bp reads. Reads from each sample were assembled using MEGAHIT. Sequencing and assembly performed by JGI.		
Data format	Raw and Analyzed		
Description of data collection	Samples were taken from seawater during cruises in 2012, six from the Arctic Polar Circle in June-July on ARK-XXVII/1 (PS80), and five from the tropical and sub-tropical Atlantic in November on ANT-XXIX/1 (PS81). Water samples were collected using 12L Niskin bottles, and seawater filtered onto 1.2-μm polycarbonate filters and frozen at − 80 °C. DNA was extracted using EasyDNA Kit as described in Martin et al [1]. Samples were snap frozen in liquid nitrogen and stored at −80 °C until sequencing. Sequencing was performed by JGI using the Illumina HiSeq platform, generating paired end 2 × 150bp reads. Assembly, gene prediction and annotation were performed by JGI IMG pipeline [2]. This pipeline identified prokaryotic MAGs, but no eukaryotes. Eukaryotic bins were subsequently identified using EukRep [3] and MetaBat [4], and genes predicted by GeneMark-ES [5] and annotated using InterProScan [6].		
Data source location	Sample name	Latitude	Longitude
	P1	79.02	-9.52
	P2	78.87	-3.23
	P3a	78.87	8.11
	P3b	78.87	8.11
	P4	73.02	9.86
	P5	71.2	8.87
	P6	69.23	7.73
	NP1	34.88	-13.14
	NP2	26.05	-17.46
	NP3	15.25	-20.52
	NP4	2.41	-13.6
	NP5	-17.28	2.98
Data accessibility	Figshare: https://doi.org/10.6084/m9.figshare.c.5017517.v4[7] Reads uploaded to NCBI SRA (https://www.ncbi.nlm.nih.gov/sra). BioProject accessions PRJNA365113, PRJNA365111, PRJNA330320, PRJNA365112, PRJNA406185, PRJNA406186, PRJNA365114, PRJNA366134, PRJNA366135, PRJNA365119, PRJNA365117, PRJNA365118.		
Related research article	Duncan, A., Barry, K., Daum, C. et al. Metagenome-assembled genomes of phytoplankton microbiomes from the Arctic and Atlantic Oceans. Microbiome 10, 67 (2022). https://doi.org/10.1186/s40168-022-01254-7[8]		

## Value of the Data

• This data spans the Arctic Circle, enabling genomic comparison of surface ocean microbes across this strong polar-temperate environmental divide.
• The eukaryotic MAGs are among the first for ocean microbes, and can be used to expand the references genomes for this group of organisms beyond the small number sequenced from cultured species.
• Can be compared to MAGs from similar environmental conditions (i.e. Antarctic) to study evolutionary responses.
• Some MAGs closely related to known species can be included in pangenomic analyses.
• MAGs which appear to display high degrees of taxonomic and functional novelty (e.g. NP3_4P, Table 1)

**Table 1:** List summarizing the MAGs available in the dataset. For prokaryotes, taxonomy was generated by GTDB-Tk [13], here both the phylum and lowest rank with a non-placeholder name is given. For eukaryotes, taxonomy is based on placement in a phylogenomic tree including protist reference genomes. Two measures of functional novelty are given: the percentage of predicted genes which lack any functional annotation, and the percentage all the Pfam domains observed which were Domains of Unknown Function. The distance between each MAG and the closest reference genome in phylogenomic trees combining MAGs and reference is given as an estimate of taxonomic novelty. Trees for eukaryotes and prokaryotes were constructed separately as detailed in the related research article, so distances are not comparable between the two. Finally, the quality of MAGs is expressed through completeness and contamination; for eukaryotes this was generated by EukCC [14], and for prokaryotes using CheckM [15].

## 1 Objective

Ocean microbes are essential for marine life, they form the base of the ocean food web and play important roles in cycling of essential nutrients. A majority of marine microbes cannot be cultured, preventing access to their genomic information through isolate sequencing and assembly methods. Metagenomics has allowed insight into the genetic material of all members of these natural communities of microbes, but to fully understand the metabolic capability and roles of individual organisms from these communities, we need to place this sequence data back into a genomic context. Binning methods for recovering MAGs have been widely applied to prokaryotes, but at the time of commencing our research we were aware of only 2 MAGs for eukaryotic marine microbes [9,10]. Our objective was to increase the range of marine eukaryotic microbes for which MAGs were available, to help better understand this environmentally significant unculturable majority. Here we describe in greater detail both the content of the repository containing MAGs and their annotation, and the methods used to produce the data.

## 2 Data Description

**Fig. 1:**
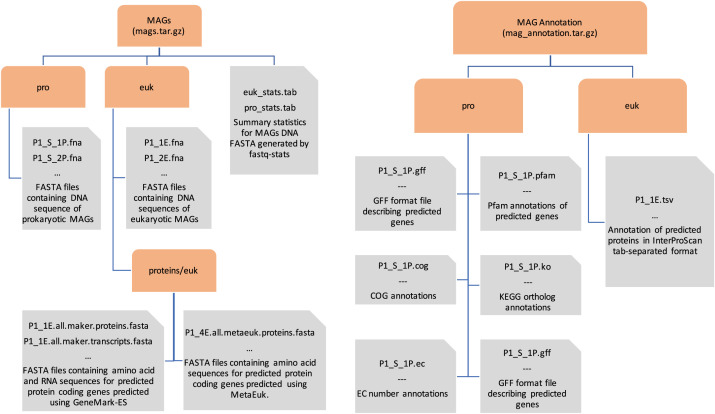
Repository structure diagram. This describes two main archives which provide the sequence data and functional annotation for the eukaryotic and prokaryotic MAGs. Tan rounded corner nodes represent directories or compressed directories, and grey nodes files. Where ellipses are included in file descriptions, this indicates that there is one such file for each MAG.

This data contains metagenome-assembled genomes, originating from samples collected in the Arctic Polar Circle and tropical and sub-tropical Atlantic Oceans. In total 143 MAGs were recovered, with 122 being prokaryotes, and 21 eukaryotes. Table 1 provides a list of all the MAGs available in the dataset. The sequence data for MAGs is the first archive making up this repository, and the annotation of the predicted genes the second. Fig. 1 shows the structure of these two components, showing directory and file structure, with more detail provided below.

**Fig. 2:**
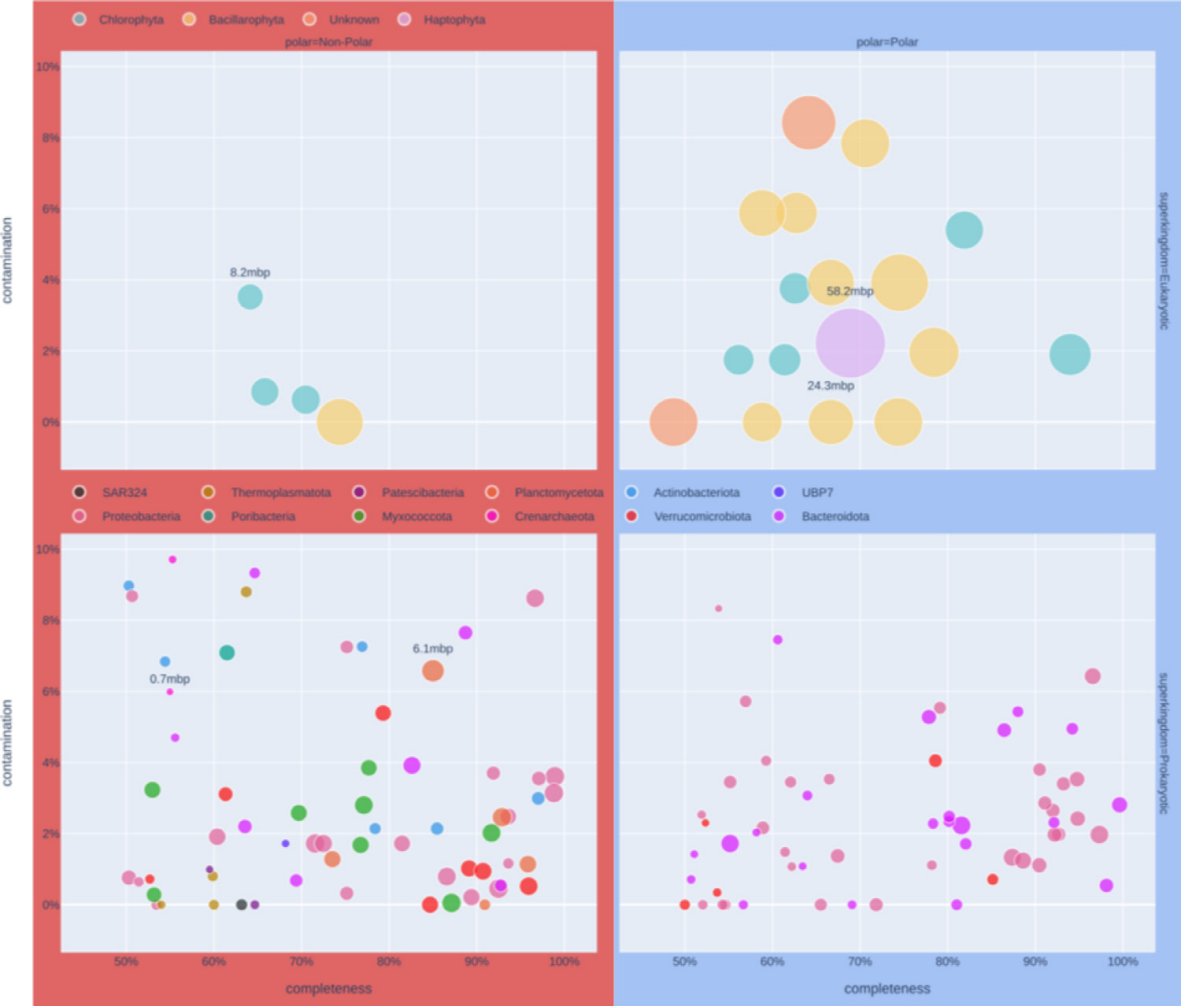
Summary of MAGs included in this dataset. Left column (red) shows MAGs recovered from non-polar assemblies, right column (blue) those within the Arctic Circle. These are further divided by domain, the top row shows eukaryotes, and the bottom row prokaryotes. Each point represents a MAG, with the size of point representing length of DNA sequence in the MAG, and the colour an estimated taxonomy. Each point is placed based MAG quality, with the horizontal axis being completeness and vertical axis contamination, assessed using EukCC [14] for eukaryotes, and CheckM [15] for prokaryotes.

**Fig. 3:**
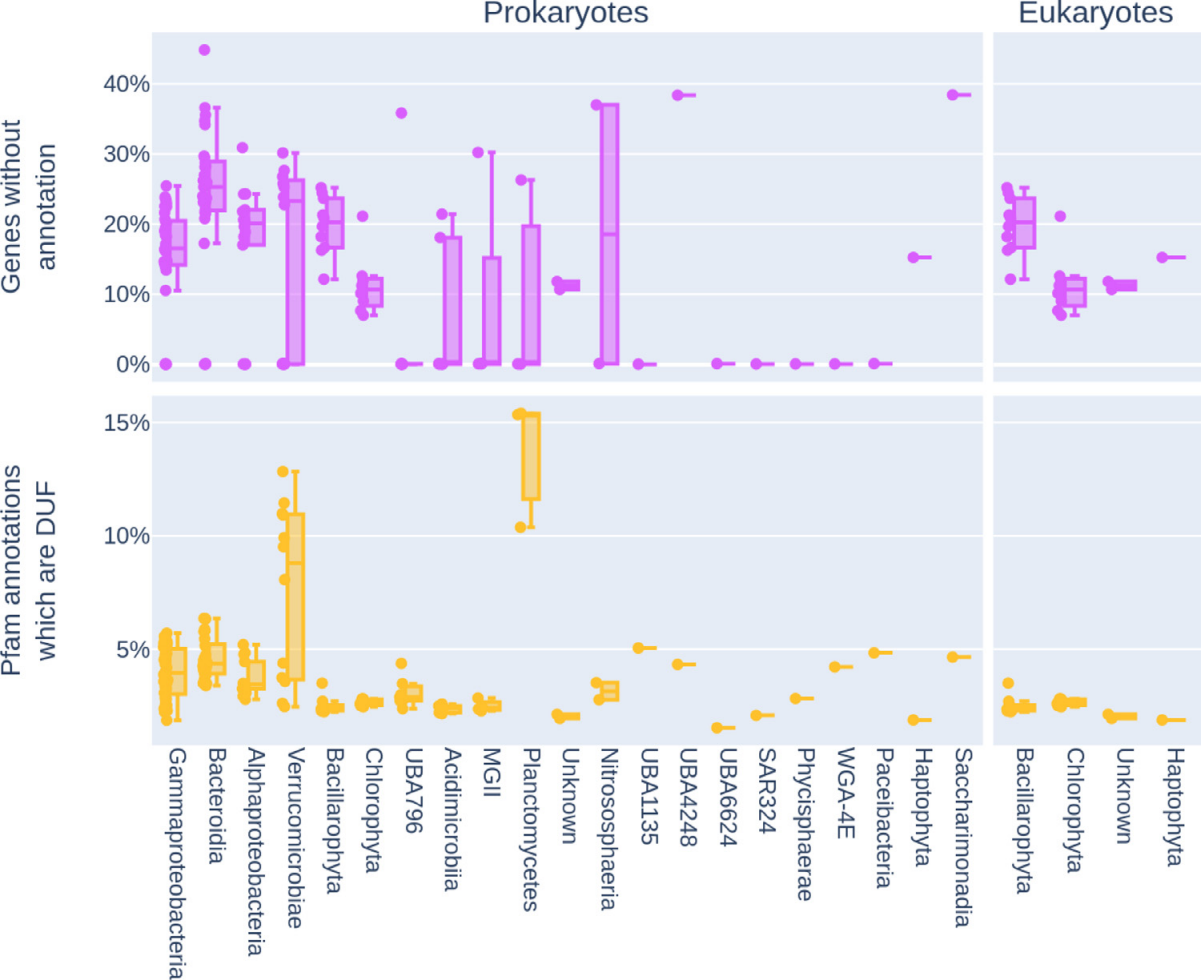
Functional novelty by phylum. The top row in pink shows the proportion of predicted genes in a MAG which had no functional annotation, shown as both a box plot and points for each MAG to the left of the box. Some phyla, such as Bacteroidia, have a high level of unannotated genes with the potential to contain functional novelty. The bottom row in orange shows what proportion of all Pfam domains in predicted genes are Domains of Unknown Function (DUF), with Planctomycetes and Verrucomicrobiae showing higher proportions of DUF domains.

143 FASTA files provide the DNA sequences for each MAG. For each eukaryotic MAG 3 files are given to describe functional annotation, and for each prokaryotic MAG 6 files are provided. Functional annotations are in different formats for eukaryotes and prokaryotes due to different tools being used for annotating them. Fig. 2 shows a summary of size, completion and taxonomy of these MAGs, with their potential functional novelty shown in Fig. 3.

For each prokaryote:

• 1 GFF file of predicted genes
• 5 tables giving annotation of genes with KEGG orthologs (KO), Enzyme Commission (EC) numbers, COG terms, Pfam domains, and a named gene product, each in tab-separated format.

For each eukaryote:

• 1 FASTA files of predicted proteins amino acid sequences
• 1 FASTA file of predicted gene transcript RNA sequences for those annotated with GeneMark-ES [5] (all but MAGs P1_4E, P1_5E, and P2_4E)
• 1 table of InterProScan [6] output in tab-separated format

These files are assigned names indicating which sample they came from, the assembler used, a numeric identifier, and whether they are eukaryotic. For example, P1_S_2P originates from sample P1, the assembler used was SPAdes [11] (rather than MEGAHIT [12]), is the 2^nd^ MAG from sample P1, and is given P for prokaryote (rather than E for eukaryote).

Hence for prokaryotes the file P1_S_2P.fna contains the contigs for this MAG, with P1_S_2P.gff the gene predictions, P1_S_2P.pfam the Pfam annotations of those genes, P1_S_2P.cog the COG annotations and so on for KOs, EC numbers, and gene products. For eukaryotes, P1_1E.fna again contains the contigs for the MAG, predicted genes are provided as their transcript and protein sequences in P1_1E.all.maker.transcripts.fasta and P1_1E.all.maker.proteins.fasta respectively, and annotation of these genes in P1_1E.tsv.

An Excel format spreadsheet contains summaries of sample and assembly details, and for MAGs their quality measures, taxonomic details, and associated metadata. The worksheets contained are:

• station_details: Information on the stations and sampling, including location, date, sampling depth, in-situ metadata including temperature, salinity, and nutrient measurements. Includes JGI and NCBI accessions for the samples.
• read_fastq_stats: Summary statistics for reads from each sample, generated by fastq-stats (length, mean quality, base frequency etc.)
• all_assembly: Summary of assembly quality for all (MEGAHIT and SPAdes) assemblies, provided by JGI.
• assembly: Same as worksheet all_assembly, but restricted to only the MEGAHIT assemblies used for eukaryotic binning
• euk_summary: The size of data at each step of eukaryotic binning. Each step gives the number of read or contigs, and the length in base pairs, for instance reads and reads_bp is the number or reads and total length of read respectively. The contigs columns give the number and length of contigs in the assembly, eukrep columns the number and length of contigs predicted as eukaryotic by EukRep, the binned columns the number and length of contigs placed in bins by MetaBat, and the mqbinned columns the number and length of contigs in medium quality bins as assessed by BUSCO.
• eukrep: Summary statistics of the predicted eukaryotic contigs, generated by BBMap.
• eukbinned: Details of the medium quality eukaryotic MAGs. Summary of sequence statistics generated by BBMap are indicated by blue columns, quality as assessed by BUSCO by red columns, and quality assessed by EukCC by yellow columns. The estimated phylum and number of predicted proteins are also given.
• pro_summary: The size of data at each step of prokaryotic binning. Columns are the same as the worksheet euk_summary, but the eukrep and binned columns are blank. EukRep was not used for prokaryote binning, and bins below medium quality were discarded by the IMG pipeline and so their size is unknown.
• pro_binned: Details of the medium quality or higher prokaryotic MAGs. Identifiers for the MAG are provided, both the name used in the repository and the Bin ID used by IMG. The column ‘Bin Quality’ contains either MQ for medium quality, or HQ for high quality. The columns in red are the quality and lineage estimated by CheckM; the usually more specific lineage from GTDB-Tk is also provided. Number and length of contigs, and number of predicted genes, are also given.
• probinned_bbmapstats: Summary statistics of the nucleic acid sequences for each MAG, generated by BBMap (number of contigs, N50, GC% etc.)
• pro_assembledby: Indicates which assembly was used for prokaryotic binning, MH being MEGAHIT, and SP SPAdes.

## 3 Experimental Design, Materials and Methods

Elven samples in total were collected for metagenome sequencing during two RV Polarstern expeditions in 2012 [1]. Samples were taken from six stations within the Arctic Polar Circle (ARK-XXVII/1 (PS80), 17^th^ June to 9^th^ July), and five from the tropical and subtropical Atlantic (ANT-XXIX/1 (PS81), 1st to 24th November). Two filtering steps were carried out, samples were first pre-filtered with a 100μm mesh to remove larger zooplankton, then filtered onto 1.2μm Nucleopore membrane filters. These were stored at − 80°C. To extract DNA, the EasyDNA Kit was used with modifications. Pre-heated (65 °C) solution A was used to wash cells off the filter, and the supernatant transferred into a new tub with a small spoon of glass beads (425–600 μm, acid-washed) (Sigma-Aldrich, USA). Samples were vortexed three times in intervals of 3s. RNAse A was added to the samples and incubated for 30 min at 65 °C. The supernatant was transferred into a new tube, and solution B from the kit was added followed by a chloroform phase separation and an ethanol precipitation. DNA was pelleted by centrifugation and washed several times with isopropanol, air-dried, and suspended in 100 μL TE buffer. DNA concentration was measured with a Nanodrop (Thermo Fisher Scientific, Waltman, MA, USA), samples snap-frozen in liquid nitrogen and stored at − 80 °C until sequencing.

Sequencing was carried out by the Joint Genome Institute, with assembly and annotation performed by their Integrated Microbial Genomes & Microbiomes (IMG/M) pipeline. The processes making up these pipelines have been published [2,16], and summarized below here.

Sequencing using the Illumina HiSeq platform generated 2 × 150bp paired-end reads. Illumina adapters were removed using BBDuk (v35.87) [17]. Subsequently reads were trimmed and filtered again using BBDuk. First read ends with quality less than 12 were trimmed. Any read pair with either three or more N characters, average quality score across the read less than 3, or length less than 51bp after trimming were discarded. Reads which map to the human HG19 genome with greater than 93% identity were also discarded, a standard part of the JGI QC pipeline. After quality control, a total approximately 629Gbp reads remained.

Quality controlled reads were assembled using MEGAHIT (v1.0.3) [12] with default parameters and a range of k-mers 23, 43, 63, 83, 103, 123. MEGAHIT assemblies contain approximately 26Gbp in 42 million contigs. The quality-controlled reads were mapped back to the assemblies to generate coverage using seal [18].

Reads from six samples (P1, P2, P3a, P6, NP3, NP5) were later reassembled using SPAdes (3.10.0-dev) [11]. This assembly used the raw unfiltered reads, which were corrected using bfc (r181) and a k-mer size of 21, then assembled using SPAdes with the meta option and range of k-mers 21, 33, 55, 77, 99, 127. The SPAdes assemblies total approximately 10Gbp and 18 million contigs. In general, the SPAdes assemblies are smaller than their MEGAHIT counterparts, but with longer mean contig lengths. Reads were mapped back to the assembly to generate overage using bwa-mem (version 0.7.15-r1142-dirty) [19] with default parameters.

Genes were predicted for each of these assemblies using an ensemble of gene prediction tools: prokaryotic GeneMark.hmm (v2.8), Prodigal (v2.6.3), MetaGeneAnnotator (August 2008), and FragGeneScan (v1.1.6) [20], [21], [22], [23]. tRNA were predicted using INFERNAL (v1.1.1) [24], and rRNA with HMMER (3.1b2) [25]; both of these need the domain as a parameter, so are run three times. Predictions from these tools are combined based on a majority consensus, with ties broken based on the predicting tool in the order they were listed above. A set of rules are applied to resolve conflicts between protein coding genes and other features (e.g. tRNA) [16]. Protein coding genes shorter than 32 amino acids are discarded.

Protein coding genes are functionally annotated with COGs, Pfams, KEGG orthologs, and EC numbers. COGs are assigned using RPS-BLAST (v2.2.31) to search against the CDD database [26,27], with an e-value cutoff of 0.1; Pfams are assigned based on search against profile HMMs using HMMER (v3.1b2) and the model specific cutoffs; KOs are assigned from LAST (737+) [28] search results against the IMG database of isolate reference genomes, and EC number based on mapping between KO and EC numbers. The best LAST hit is used to assign taxonomy to the gene, and the taxonomy of contig is the lowest common ancestor of all the genes on the contig, where 30% or greater of the genes have any LAST hits. A total of approximately 50 million genes were predicted.

The binning process incorporated into the IMG/M pipeline identified 122 prokaryotic MAGs. Each assembly was binned individually using MetaBat (v2.12.1) [4] using a minimum contig size of 3000bp, coverage of the contigs in samples other than the one the assembly was generated from was not used. Quality of bins were assessed using CheckM (v1.0.12) [15], and only medium quality bins were retained (≥50% completeness, ≤10% contamination). Taxonomy of MAGs was assessed with GTDB-Tk (v0.2.2, database release 86) [13]. These MAGs are available both in this repository, and on the IMG website using the bin identifiers included in the summary spreadsheet.

MAGs identified by the IMG/M pipeline were all prokaryotic, prompting a separate binning effort to recover eukaryotes. Only the MEGAHIT assemblies were used for eukaryotic binning. Eukaryotic contigs in were identified in each assembly using EukRep (v0.6.5) [3] with default parameters, producing a total of approximately 4Gbp and 2 million eukaryotic contigs. To estimate the coverage of these eukaryotic contigs in all samples, reads from each sample were pseudo-aligned to each of the 12 sets of eukaryotic contigs using the Kallisto (v0.44.0) [29] kallisto-quant command with default parameters. The estimated mean coverage of each contig was taken to be the number of reads estimated to originate from that contig multiplied by the read length (150bp) divided by the length of the contig. This was formatted into a table for each set of eukaryotic contigs, with the contig as rows, set of reads as columns, and each entry the estimated coverage. Binning was performed for each set of eukaryotic contigs with MetaBat (v2.12.1) with this coverage information as input and a minimum contig size of 1500bp, and otherwise default parameters. This produced 59 bins; to match the prokaryotes the quality of these bins was assessed using BUSCO (v3.0.2) [30] and the eukaryota_odb9 set of genes, and only the 18 MAGs which were medium quality or better retained.

Although genes had been predicted on all contigs by the IMG/M pipeline, this had been using tools which were not adapted to the more complex gene structure of eukaryotes. Hence, genes were predicted for these 18 eukaryotic MAGs using MAKER (v2) [31] and GeneMark-ES (v4.38) [5] in self-training mode. A GeneMark-ES model was trained using gmes_petap.pl command with the MAG contigs as input with a minimum contig length of 5000bp. The resulting model was used by MAKER with otherwise default parameters. GeneMark-ES has the assumption that all contigs originate from a single genome, so gene prediction had to be carried out after binning for these eukaryotic MAGs.

After this initial eukaryotic binning effort, colleagues at JGI identified 3 additional eukaryotic bins (P1_4E, P1_5E, and P2_4E) using alternative methods. Starting with the assemblies, contigs were searched against the Marine Microbial Eukaryote Transcriptome Sequencing Project (MMETSP) [32] database using MMSeqs2 [33] to filter for eukaryotic contigs. These were each binned using Metabat (v2.12.1), and resulting bins checked for taxonomic consistency using the MMSeqs2 results. Any bin with 50% or greater contigs from a single phylum and total length 5Mbp or greater was retained, and filtered to remove contigs from other taxa. This resulted in three additional MAGs, for which genes were predicted using MetaEuk [34] with NR [35] used as reference database. These three additional MAGs were added to the repository.

Completeness and contamination of these 21 eukaryotic MAGs was assessed using EukCC (v0.2) [14] to obtain lineage specific estimates of quality. For these eukaryotic MAGs, the predicted proteins were annotated using InterProScan (v5.37-75.0) [6] with default parameters.

## Ethics Statements

The authors have consulted the publishers Ethics in Publishing standards, and believe the manuscript meets these standards.

## CRediT authorship contribution statement

**Anthony Duncan:** Investigation, Formal analysis, Writing – original draft, Visualization. **Kerrie Barry:** Investigation. **Chris Daum:** Investigation. **Emiley Eloe-Fadrosh:** Investigation. **Simon Roux:** Investigation, Formal analysis, Writing – review & editing. **Katrin Schmidt:** Investigation. **Susannah G. Tringe:** Investigation, Formal analysis. **Klaus U. Valentin:** . **Neha Varghese:** Investigation, Formal analysis. **Asaf Salamov:** Investigation, Formal analysis, Writing – review & editing. **Igor V. Grigoriev:** Investigation, Formal analysis, Writing – review & editing. **Richard M. Leggett:** Conceptualization, Writing – review & editing, Supervision. **Vincent Moulton:** Conceptualization, Writing – review & editing, Supervision. **Thomas Mock:** Conceptualization, Writing – review & editing, Supervision.

## Declaration of Competing Interest

The authors declare that they have no known competing financial interests or personal relationships that could have appeared to influence the work reported in this paper.

## Data Availability

Metagenome Assembled Genomes of 21 eukaryotic and 122 prokaryotic phytoplankton, with predicted proteins and functional annotation (Original data) (NCBI BioProject). Metagenome Assembled Genomes of 21 eukaryotic and 122 prokaryotic phytoplankton, with predicted proteins and functional annotation (Original data) (NCBI BioProject). Metagenome Assembled Genomes of 21 eukaryotic and 122 prokaryotic phytoplankton, with predicted proteins and functional annotation (Original data) (NCBI BioProject). Metagenome Assembled Genomes of 21 eukaryotic and 122 prokaryotic phytoplankton, with predicted proteins and functional annotation (Original data) (NCBI BioProject). Metagenome Assembled Genomes of 21 eukaryotic and 122 prokaryotic phytoplankton, with predicted proteins and functional annotation (Original data) (NCBI BioProject). Metagenome Assembled Genomes of 21 eukaryotic and 122 prokaryotic phytoplankton, with predicted proteins and functional annotation (Original data) (NCBI BioProject). Metagenome Assembled Genomes of 21 eukaryotic and 122 prokaryotic phytoplankton, with predicted proteins and functional annotation (Original data) (NCBI BioProject). Metagenome Assembled Genomes of 21 eukaryotic and 122 prokaryotic phytoplankton, with predicted proteins and functional annotation (Original data) (NCBI BioProject). Metagenome Assembled Genomes of 21 eukaryotic and 122 prokaryotic phytoplankton, with predicted proteins and functional annotation (Original data) (NCBI BioProject). Metagenome Assembled Genomes of 21 eukaryotic and 122 prokaryotic phytoplankton, with predicted proteins and functional annotation (Original data) (NCBI BioProject).
